# Polymer Composite-Based Materials with Photocatalytic Applications in Wastewater Organic Pollutant Removal: A Mini Review

**DOI:** 10.3390/polym14163291

**Published:** 2022-08-12

**Authors:** Alexandru Enesca, Cristina Cazan

**Affiliations:** Product Design, Mechatronics and Environmental Department, Transilvania University of Brasov, Eroilor 29 Street, 35000 Brasov, Romania

**Keywords:** advanced oxidation, composites, dyes, organic pollutants, pharmaceutical compounds, photocatalysts

## Abstract

The development of new technologies using nanomaterials has allowed scientists to design advanced processes with many applications in environmental protection, energy production and storage, and medicinal bio-mediated processes. Due to their significant potential applications in different branches of science, the development of new polymer composites represents a priority, especially for nano-technological processes. Interest in polymeric composites was outlined by the synthesis of a large number of nano- or mezzo-scale materials with targeted functional properties for polymer matrix hybridization. The present mini review explores some of the most representative and recent papers reporting the photocatalytic activity of polymer composites toward different organic compounds (dyes, pharmaceutically active molecules, phenol, etc.). The polymer composites were divided based on their composition and photocatalytic activity. TiO_2_- and ZnO-based polymeric composites have been described here in light of their photocatalytic activity toward different pollutants, such as rhodamine B, phenol, or methyl orange. Polymeric composites based on WO_3_, Fe_2_O_3_, or Bi_2_MoO_6_ were also described. The influence of different polymeric composites and photocatalytic parameters (light spectra and intensity, pollutant molecule and concentration, irradiation time, and photocatalyst dosage) on the overall photocatalytic efficiency indicates that semiconductor (TiO_2_, ZnO, etc.) insertion in the polymeric matrix can tune the photocatalytic activity without compromising the structural integrity. Future perspectives and limitations are outlined considering the systematic and targeted description of the reported results. Adopting green route synthesis and application can add economic and scientific value to the knowledgebase by promoting technological development based on photocatalytic designs.

## 1. Introduction

The need for available clean and drinking water has significantly increased due to demographic expansion, technological development, and higher comfort standards [1]. These factors put an immense stress on the clean water scarcity which emerges as a global problem threatening human health and future lifestyle. In 2019, the World Health Organization released a report indicating that around 785 million people have no or limited access to drinking water and more than half to the Earth population will encounter drinking water scarcity by 2025 [2,3].

The development of new technologies using nanomaterials has allowed scientists to design advanced process with many applications in environmental protection, energy production and storage, as well as medicinal bio-mediated processes. Due to their significant potentials applications in different branches of science, the development of new polymer composites represents a priority, especially for nano-technological processes [4,5]. Polymers based on nano-composites are considered as hybrid structures composed of organic or inorganic nano-materials coupled with polymers acting as matrices with different sizes and shapes. Such nano-composite structures exhibit distinctive chemical and physical properties, which cannot be attributed to a stand-alone component and are a consequence of the effects of synergistic dual components. Interest in polymeric composites was outlined by the synthesis of a large number of nano- or mezzo-scale materials with targeted functional properties for polymer matrix hybridization. These hybrid materials can be used in different fields, such as organic pollutant removal, energy production and storage, catalytic assisted processes, etc. [6,7,8]. Polymer-based nanomaterials have been used in the medical field, especially in the total hip implant to improve the mechanical performance of polymers [9].

Persistent organic pollutants (POPs) can be found in many aquatic bodies due to direct release or indirect contamination, and the concentration varies depending on the pollution source. The traditional processes for wastewater treatment are inefficient for the complete removal of POPs and advanced technology is required to address this issue [10]. Bioaccumulation has the disadvantage of moving the pollutant from one site to another, while the main issue remains unsolved. Fenton-based processes use light and cations as driving forces for pollutant removal, but the by-product formation is an important disadvantage. The advanced oxidation process (AOP) is considered as a promising alternative for the complete decomposition of POPs due to its ability to generate oxidative and super-oxidative species when a catalyst is irradiated with different sources (microwave, solar radiation, ultraviolet (UV), visible (vis), or other variants) [11,12]. Mono-component semiconductor metal oxides (such as WO_3_ [13,14], TiO_2_ [15,16], ZnO [17,18], SnO_2_ [19,20], MnO_2_ [21,22], etc.) and heterostructures (such as ZnO/CuO [23,24], TiO_2_/ZnO [25,26], Cu_2_S/WO_3_ [27,28], TiO_2_/WO_3_/ZnO [29], Cu_2_S/WO_3_/SnO_2_ [30], CuO/ZnO/WO_3_ [31], etc.) have been intensively studied and characterized as suitable candidates for photocatalytic wastewater treatments. However, these materials raise significant issues in large-scale applications due to their limited absorption range, surface warping, interface chemical stability, charge carrier recombination, and mobility [32,33,34].

Polymeric composites are considered as suitable candidates for AOPs due to their unique physical and chemical properties induced by the formation of the interphase region, fillers, and matrix. Polymeric materials are widely used in the industry, due to their lightweight, versatile processability, high chemical resistance, low cost, and low specific gravity [35,36]. The use of conductive polymers as sensitizing agents in photocatalytic composites have the advantage of improving the photocatalytic efficiency due to p-conjugated systems which contain a high concentration of electron-rich species available for transfer in the semiconductor’s conduction band. Consequently, the recombination rate of the photo-generated charge carriers is reduced, while conductive polymers tune the semiconductor’s band gap [37,38]. Based on the density functional theory models, the conducting polymers such as poly(1-naphthylamine), polythiophene, polyaniline, polyacetylene, polypyrrole, polycarbazole, or poly(ophenylenediamine) exhibit visible absorption activity due to their low band gap values which make them ideal candidates for extending the semiconductor’s light absorption range [39,40,41,42]. A systematic review can be used as a tool on designing new experiments to provide more insight on the correlation between the composite composition, testing parameters, and overall photocatalytic activity.

The present mini review explores some of the most representative and recent papers reporting on the photocatalytic activity of polymer composites toward various organic compounds (dyes, pharmaceutically active molecules, phenol, etc.). The literature contains scientific articles presenting similar or different information that was not considered here due to the space limitation or incomplete experimental data. The paper describes the effect of different polymeric composites and photocatalytic parameters (light spectra and intensity, pollutant molecule and concentration, irradiation time, photocatalyst dosage, etc.) on the overall photocatalytic efficiency. Representative studies were included and corelated in order to outline the significance of polymeric composite composition and testing parameters on the photocatalytic removal of pollutants. It can be seen that high light intensity or photocatalyst dosage are not pre-requisites for high photocatalytic activity. Tailoring the material properties based on their composition as well as the surface chemistry with the pollutant molecules can play an important role.

## 2. Photocatalytic Mechanism of Polymeric Composites

Nanotechnology may represent an alternative solution in solving the issues related to safe drinking water stress and high global demography. The available drinking water sources are inconsistent with the geographical position of high urban density, and so long-term solutions are needed. As a flexible alternative for cost-effective wastewater treatment processes, nanotechnology exhibits high efficiency and multifunctionality and is suitable for large-scale applications [43,44]. Additionally, the photocatalytic process using nanomaterials can integrate renewable resources in the technological wastewater treatment flux as a key component of energy sustainability. The main goal of the photocatalytic process is to induce the pollutant mineralization, leading to non-hazardous products. However, the polymer composite requires large optimization procedures in order to attempt this goal. Moreover, the pollutant is partially decomposed, which may lead to hazardous by-products [45,46].

**Figure 1:**
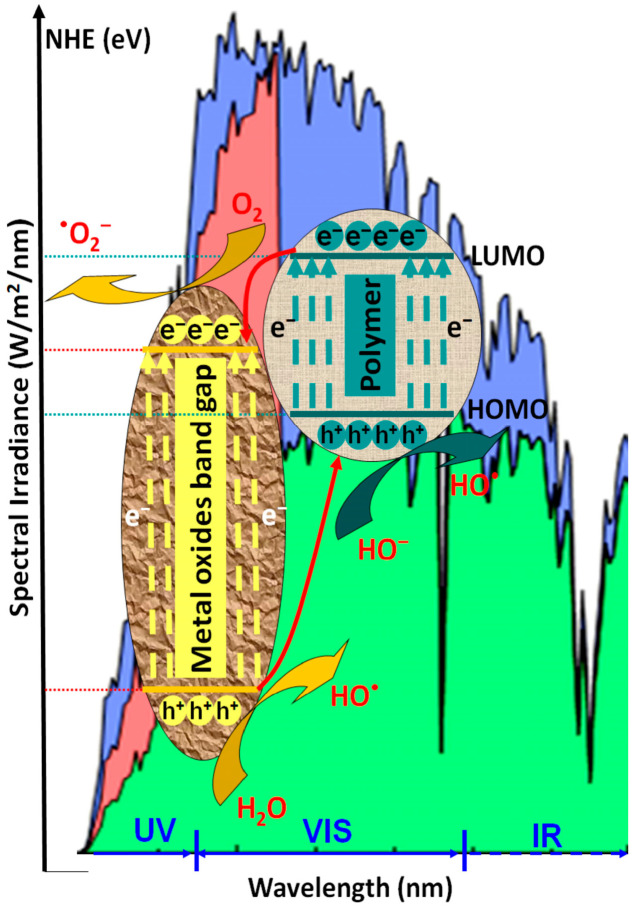
The photocatalytic mechanism of polymeric composites.

Photocatalysts based only on metal oxide semiconductors present limitations in terms of charge carrier recombination and mobility, chemical stability, and large band-gaps values, which restrict the light absorption spectrum in the UV region [47,48,49]. As presented in Figure 1, when coupled with metal oxide semiconductors, the conducting polymers work as photosensitizers due to their high charge mobility properties.

The polymeric composite exhibits a high-efficiency response in the presence of vis light radiation, thermal and chemical stability, and low charge carrier recombination [49]. During irradiation (*hν*), the composite interface behaves as a synergic active area where the photogenerated electrons (e^−^) migrate from the lowest unoccupied molecular orbital (LUMO) polymer level to the metal oxide conduction band (CB), while the photo-induced holes (h^+^) are transferred from the valence energy band (VB) of metal oxide directly on the highest occupied molecular orbital (HOMO) polymer level [50,51]. As presented in Equations (1)–(9), the photocatalytic inter-chain reaction is induced by the formation of hydroxyl radicals (•OH) from water molecules along with the formation of superoxide anion radicals (•O_2_^−^) from oxygen-dissolved molecules. Both mechanisms require the participation of photogenerated charge carriers (h^+^ and e^−^). The subsequent reactions of •O_2_^−^ with photogenerated holes can form hydroperoxyl radicals (•OOH) and H_2_O_2_ [52]. It must be underlined that the direct oxidation of organic pollutants absorbed at the photocatalyst surface is also possible [53].

(1) $$\mathrm{Polymeric}\;\mathrm{Composite}+h\upsilon \to e^{-}+h^{+},$$

(2) $$H_{2}O+h^{+}\to {\mathrm{HO}}^{-}+H^{+},$$

(3) $${\mathrm{HO}}^{-}+h^{+}\to \mathrm{HO}•,$$

(4) $$O_{2}+e^{-}\to •O_{2}^{-},$$

(5) $$•O_{2}^{-}+H^{+}\to •\mathrm{OOH},$$

(6) $$•\mathrm{OOH}+•\mathrm{OOH}\to H_{2}O_{2}+O_{2},$$

(7) $$H_{2}O_{2}+e^{-}\to •\mathrm{OH}+{\mathrm{HO}}^{-}$$

(8) $$\mathrm{Organic}\;\mathrm{Molecule}+•\mathrm{OH}/•O_{2}^{-}\to \mathrm{Degradation}\;\mathrm{By-products}$$

(9) $$\mathrm{Degradation}\;\mathrm{By-products}+•\mathrm{OH}+•O_{2}^{-}+h^{+}\to \mathrm{Mineralization-products}$$

## 3. Polymer Composite Photocatalytic Materials

Despite the advantages of polymeric materials, their low modulus, low strength, low working temperature, and chemical stability to environmental conditions limit their application in photocatalytic processes. The insertion of a secondary material, such as metals or metallic oxides, as a reinforcement component can provide a unique combination of optical and electrical properties which will enhance photocatalytic activity. Furthermore, the resulting composites possess superior properties compared with stand-alone components, including improved chemical resistance, modulus, strength, and electrical conductivity [54,55].

### 3.1. Polymer/TiO_2_- or ZnO-Based Composites

**Figure 2:**
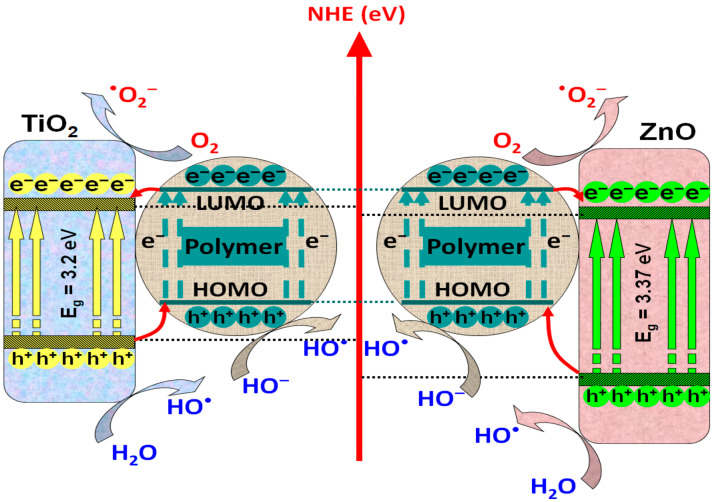
Mechanism of charge photogeneration in polymers/TiO_2_ or ZnO composites.

Among transition metal oxides, titanium oxide is considered as the most widely used semiconductor due to its high chemical stability, versatile morphology, and high UV sensitivity; however, in recent years, ZnO has earned considerably attention because of its catalytic properties and cost-effective synthesis procedures [56]. As presented in Figure 2, both semiconductors can be coupled with polymers in order to form favorable junction for the photogeneration and transfer of charge carriers, in order to produce oxidative species. Table 1 contains representative studies of polymers/TiO_2_ or ZnO composites used in photocatalytic applications.

The photocatalytic removal of methyl orange was evaluated using composites based on polyacrylamide/TiO_2_ [57], polyurethane/TiO_2_ [58], and resorcinol-formaldehyde/TiO_2_ [59]. The polyurethane/TiO_2_ could completely remove the methyl orange dye in 50 min from a solution with a 10 mg/L concentration using a catalyst dosage of 0.046 g/3.6 mL. The composite has the advantage of using macroporous supports for TiO_2_ insertion which favor the formation of a larger active surface. Consequently, the photo-oxidation rate increases and the mass transfer is reduced. The polyacrylamides/TiO_2_ exhibit 95% photocatalytic efficiency after 300 min of UV irradiation with a 3 W light source. The initial concentration of methyl orange solution was significantly lower (1.0 mg/L), which allows higher light penetration during the experiments. The diffusion plays an important role in the photocatalytic efficiency. At low diffusion values, the dye concentration decreases more slowly with time on the outer boundary compared with the case of the fast diffusion. The highest methyl orange concentration (13 mg/L) was used to test the resorcinol-formaldehyde/TiO_2_ photocatalytic activity at a 1 mg/3 mL dosage. The sample was irradiated with a 36 W UV lamp for 240 min in order to remove 55% of methyl orange. A lower photocatalytic activity was presumed to be induced by the TiO_2_ influence which reduces the apparent polymeric surface, in turn decreasing the number of active sites involved in dye degradation.

The photocatalytic removal of methylene blue was tested using polyethylene glycol/TiO_2_ [60], polytrifluorochloro ethylene/TiO_2_ [61], and PEG2000–silicone/TiO_2_ [62] composites. Both polyethylene glycol/TiO_2_ and polytrifluorochloro ethylene/TiO_2_ could completely remove the dye, but under different experimental conditions. The polytrifluorochloro ethylene/TiO_2_ composite was tested using a 10 mg/L methylene blue concentration and 270 min of UV irradiation (0.5 mW/cm^2^). The overall film roughness was improved due to the TiO_2_ nanopowder embedded in the polymer matrix, which maximizes the interface with solution. When roughness increases, these may suggest that the TiO_2_ nanoparticles form aggregates, which can reduce the photocatalytic activity. The concentration of methylene blue solution was double (20 mg/L) for the polyethylene glycol/TiO_2_ photocatalytic test and the composite could remove the dye in 240 min under a 300 W UV source using a catalyst dosage of 3 g/100 mL. It is well known that the surface morphologies play an important role in dye adsorption, which is the preliminary step for their degradation. Variations in adsorption capacity were observed due to the PEG increasing the porosity, resulting in an increased interface area with the dye solution. However, when a mixture of PEG2000–silicone/TiO_2_ was used, the photocatalytic efficiency decreases at 40%, even if the initial concentration of methylene blue solution was limited at 3.2 mg/L and the UV exposure time was 120 min. In this case, the risk of mechanical collapse was reduced due to the presence of surface hydroxyl groups, allowing the particles to bond tightly to the modified silicone.

The TiO_2_ was employed to form composites with polyvinyl alcohol and polyethylene glycol for the photocatalytic removal of acid black dye in similar experimental conditions [63]. The dye solution concentration was 20 mg/L and the catalyst’s dosage was 50 mg/400 mL. After 120 min of UV irradiation with 44 W/m^2^ irradiance, the polyvinyl alcohol/TiO_2_ exhibits 55.4% photocatalytic efficiency, while polyethylene glycol/TiO_2_ reaches 62.8% efficiency. It was found that the polyethylene glycol/TiO_2_ composite showed higher photocatalytic activity due to the more appropriate calcination temperature, allowing the interaction of Ti-O-C bonds in the structure. Rhodamine B dye photodegradation was also tested with the polythiophene/TiO_2_ composite using UV (10 W) and vis (320 W) radiation [64]. The dye solution concentration was 40 mg/L and the catalyst dosage was 300 mg/300 mL. The results indicate that after 180 min of UV radiation, 76% of rhodamine B was removed, while under vis radiation, 600 min were necessary to remove 98% of rhodamine B. Even if the TiO_2_ is absorbed in the UV region, surface hybridization in the presence of polythiophene can induce visible light photon absorption. The removal of rhodamine B was also tested with trans-anethole/N-phenylmaleimide/TiO_2_ composite poly-phenylpropenes under 600 W UV irradiation using a catalyst dosage of 20 mg/20 mL [65]. After 90 min of irradiation, 95% of the 10 mg/L dye solution concentration was removed. The number of free radicals photogenerated under UV irradiation and the available number of adsorption sites were limited as the dye could saturate a part of them. Increasing the irradiation time can help in generating more oxidative radicals involved in rhodamine B degradation, but the energy consumption will also increase. The same composite and experimental conditions were used for the removal of tetracycline. In this case, the photocatalytic efficiency increased at 97%, indicating that trans-anethole/N-phenylmaleimide/TiO_2_ poly-phenylpropenes are versatile composites that can be used to remove different organic pollutant. Another pharmaceutical compound (metronidazole) was considered for photocatalytic degradation using the chitosan polyvinyl alcohol/TiO_2_ composite [66] with a 0.3 g/L dosage. The metronidazole was completely removed from the 10 mg/L solution concentration in 120 min under 32 W UV radiation based on the synergetic effect of pseudo-second-order adsorption and pseudo-first-order photocatalytic processes. Density functional theory modeling (DFT) indicates that the assembly pf hydroxyl groups will passivate the titanium oxide to the polymer through significant hydrogen bonding. The adsorption of metronidazole molecules on the composite surface is favored by the extensive weak dispersive forces.

Phenol photocatalytic removal was evaluated using polyvinyl alcohol/TiO_2_ [67] and poly(vinylidene fluoride)/GO/TiO_2_ [68] composites. A visible light source with a 500 W intensity was used to irradiate polyvinyl alcohol/TiO_2_ for 360 min. The results indicate that 67.5% of the phenol was removed to form a 10 mg/L solution due to the short diffusion length of excitons, which can induce their dissociation prior recombination. The employment of conjugated PVA will improve the TiO_2_ absorption, resulting in higher energy conversion. The dissociation of charge carriers can promote the charge migration from the polymer to the TiO_2_ nanoparticles. The poly(vinylidene fluoride)/GO/TiO_2_ composite was irradiated with a 100 W UV source for 180 min. The photocatalytic efficiency was 65%, but the initial phenol concentration was five times higher than in the previous case. This is due to the GO presence which serves as an electron receptor and network for the photo-generated charges in TiO_2_. The photo-generated charged carriers migrate through the GO sheets that reduce the electron–hole recombination due to the stereo conductive framework which can alleviate the recombination rate process. The removal of three types of parabens (methylparaben, ethylparaben, and propylparaben) was performed by placing the polydimethylsiloxane/TiO_2_ composite under sun radiation for 120 min [69]. Using the same paraben concentration (1 mg/L) and photocatalyst dosage (140 mg/L), the removal efficiency varies up to 50% for methylparaben, 52% for ethylparaben, and 55% for propylparaben. The authors indicate that TiO_2_, when immobilized in the polymeric membranes, is the only component acting for the removal of parabens through the photocatalytic process since no adsorption on the polymeric membranes occurs. Moreover, increasing the TiO_2_ concentration favors the nanoparticle agglomeration, which can inhibit the TiO_2_ active sites and decrease the available active centers responsible for hydroxyl radical production.

**Figure 3:**
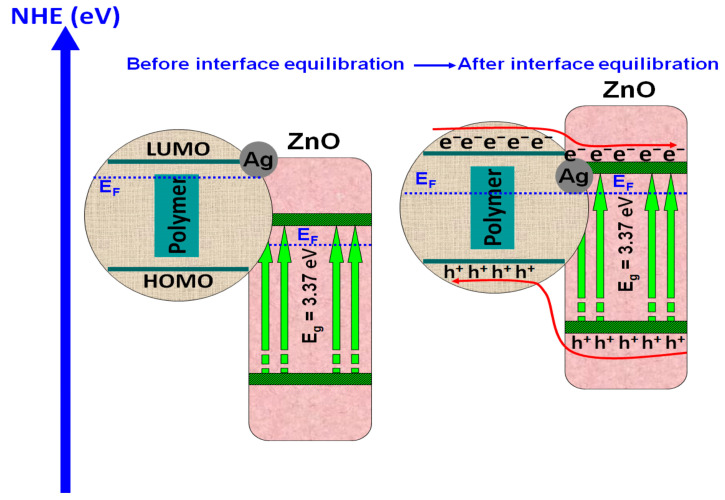
Polymeric composite containing silver coupled ZnO.

Polymeric composites with ZnO were employed for the photocatalytic removal of dyes such as methylene blue [70], rhodamine B [71,72], or methyl orange [73]. The polypyrrole/ZnO composite was used to remove methylene blue dye from a 50 mg/L concentrated solution [70]. After 20 min of 100 W UV irradiation and a 50 mg/50 mL catalyst dosage, the photocatalytic efficiency was 98.12% due to the suitable charge carrier transfer mechanism between polypyrrole and ZnO nanoparticles that reduce the charge recombination. The study indicates that, at higher temperatures (323 K), photocatalytic efficiency increases at 99.05%, which shows that the degradation process follows an endothermic pathway. A similar composite based on polypyrrole/ZnO was used for rhodamine B photocatalytic removal with a 5 mg/L solution concentration [71]. The sample was irradiated for 300 min with a 150 W vis radiation, and the final photocatalytic efficiency was 65%. During the composite light irradiation, excitons were created. The photo-generated electron in ZnO was transferred from the valence energy band to the CB, and the photo-generated hole migrated to highest occupied molecular orbital (HOMO) of the polymer. Additionally, the photo-generated electron from HOMO migrated to lowest energy unoccupied molecular orbital (LUMO) in the polymer matrix. Finally, from LUMO, the photo-generated electron was transferred to the conduction band of ZnO. When the poly(3-hexylthiophene-2,5-diyl)/ZnO composite was used to remove 0.01 mg/L rhodamine B under 300 W vis radiation, the photocatalytic efficiency increased by 99% after 80 min of irradiation [72]. This result can be attributed to the strengthened vis light absorption and the closely contacted interface between the two components. A comparative study for methyl orange photocatalytic removal was conducted using poly(propylene glcol)-dimethacrylate/methacryloyloxyethyl-N,N-dimethyl-3-(trimethoxysilyl)-propane/ZnO and poly(propylene glycol)-dimethacrylate/methacryloyloxyethyl-N,N-dimethyl-3-(trimethoxysilyl)-propane/ZnO-Ag composites [73]. The samples were irradiated with vis light (4.9 mW/cm^2^) for 250 min in order to remove 16.35 mg/L of methyl orange. The silver-free composite exhibited 56.12% photocatalytic efficiency, while the methacryloyloxyethyl-N,N-dimethyl-3-(trimethoxysilyl)-propane/ZnO-Ag composite reached 95% efficiency. As presented in Figure 3, a lower ZnO-Ag Fermi level relative to the ZnO conduction band energy was established during irradiation, inducing the photo-generated migration of electrons from ZnO to the silver-mediated composite using the potential energy. Consequently, the accumulation of photo-generated electrons occurred in silver particles along with the hole’s migration to ZnO surface, inducing the charge carrier’s separation. The insertion of silver nanoparticles had a favorable effect on the generation of oxidative radicals, where electrons were adsorbed by the O_2_ to form •O_2_^−^ and holes reacted with OH^−^ to form hydroxyl radicals (•OH).

### 3.2. Other Polymer Composites Based Materials

Polymers can be combined with one or more components in order to form suitable composites for photocatalytic applications. The components are chosen based on several factors: interface compatibility with the polymeric matrix, chemical stability in the working environment, light sensitivity, and economic costs. Table 2 presents some recent reports on polymeric composites containing tandem semiconductors, metals, or mono-component semiconductors with photocatalytic application for dyes or the removal of pharmaceutically active compounds.

#### 3.2.1. Polymer/Tandem Structure-Based Materials

**Figure 4:**
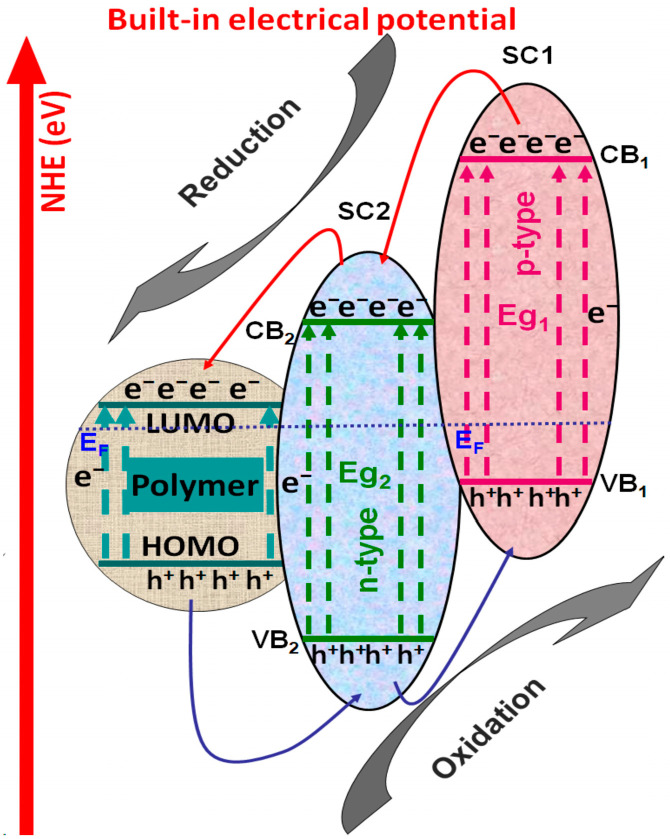
Photocatalytic composite based on polymer/tandem materials.

Tandem structures are composed of at least two semiconductors with suitable disposal of the energy levels, allowing the mobility of charge carriers through the structure and reducing the charge recombination rate. As presented in Figure 4, the tandem structure can be combined with conductive polymeric materials in order to enhance the charge photo-generation, favoring the reduction reactions with e^−^ and oxidation reactions by h^+^. These charge carriers will be involved in the oxidative species development, responsible for pollutant mineralization. The non-conductive polymeric matrix can be used to increase the light absorption spectrum, tandem particle dispersion, and the specific surface.

The photocatalytic removal of three dye molecules (methylene blue, crystal violet, and congo red) was studied using the poly(vinyl alcohol-g-acrylamide)/ZnO/SiO_2_ composite [74]. The dye’s concentration was established at 5 mg/L and the photocatalyst dosage was 0.1 g/20 mL. After 960 min of irradiation with a 18 W UV source, the removal efficiencies were 86% for methylene blue, 77% for crystal violet, and 70% for congo red. The photocatalytc removal efficiency depends on the surface chemistry between the photocatalyst and the dye molecules. When the electrostatic interaction, repelling force, driving force, and hydrogen bonding between dye molecules and composite active sites are in equilibrium, the saturated state facilitates the photocatalytic mineralization reactions. The main driving force for this process is represented by the mass transfer of dye molecules from solution that could reach the available composite active surface sites.

The total removal of congo red was reached with the polyaniline/Cu_2_O/ZnO composite, using a 30 mg/L dye concentration and a 100 mg/100 mL catalyst dosage [75]. Half an hour was enough to remove the dye due to the insertion of an oxidizing promoter based on ammonium persulfate/potassium permanganate. The composite performance was attributed to the small crystallite sizes (18.5 nm), low bandgap (2.68 eV), reduced and low PL intensity, and large surface area (45.32 m^2^/g). The high photocatalytic removal of methylene blue was reported on polysulphone–styrene maleic anhydride copolymer/Bi_2_S_3_/TiO_2_ composites [76]. The catalyst was irradiated for 180 min with a UV–vis (350 W) source and 95% of the 20.25 mg/L dye solution concentration was removed. The composite absorption includes the entire visible-light spectra, due to the Bi_2_S_3_’s small band gap value. Bi_2_S_3_ is a direct transition n-type semiconductor with a 1.3 eV band gap energy, which can use the complete visible spectra and enhance the composite light absorption spectrum. The narrower band gap energy of the Bi_2_S_3_-TiO_2_ tandem structure stimulates the photo-electric process required to induce the charge carrier’s generation. The combined effect of adsorption–migration–photodegradation occurs in the molecular interfacial layers. The reactant’s surface and photocatalytic affinity adsorption properties are significant in determining the overall reaction rate.

Rhodamine B photocatalytic removal was evaluated using polyvinyl chloride/Ag-decorated Bi_2_O_3_/Bi_2_O_2.7_ [77] and polypyrrole/TiO_2_/V_2_O_5_ [78] composites. The polyvinyl chloride/Ag-decorated Bi_2_O_3_/Bi_2_O_2.7_ exhibit 97% photocatalytic efficiency after 150 min of irradiation with a low-intensity vis source (5 W). The photocatalytic activity was attributed to the •O_2_^−^ and h^+^ photo-generation based on the synergistic effects of good interface quality; petal-like hierarchical shape; and fast charge separation between Ag particles, β-Bi_2_O_3_, and Bi_2_O_2.7_. If the composite uses the same composition on each side of the heterostructures surface with good lattice fringes matching, then it can establish a suitable and tuned channel for the charges produced during the irradiation. This advantage originates from lower spacing lattice distances between the catalyst partners that may induce lower lattice mismatch, and subsequently decrease the number of defects and interface penetration barriers. The use of the circular band structure provides higher photo-generated carrier migration and separation, due to the minor and potential differences between components. The second composite based on polypyrrole/TiO_2_/V_2_O_5_ can completely remove the rhodamine B dye after 120 min of irradiation with a 300 W vis source. The same composite was employed to establish the photocatalytic performance toward four molecules of pharmaceutically active compounds. Using a 50 mg/L solution concentration and a 30 mg/50 mL photocatalyt dosage, the composite exhibits the following removal efficiencies: 98% for tetracycline, 96% for doxycycline, 85% for oxytetracycline, and 37% for ofloxacin. The presence of V_2_O_5_ increases the vis light absorption in the 600 nm proximity. The introduction of polypyrrole remarkably broadens the composite absorption region to the total visible light spectrum, which simultaneously enhances the light absorption performance and photocatalytic activities under visible light.

#### 3.2.2. Polymer/Metal-Based Materials

**Figure 5:**
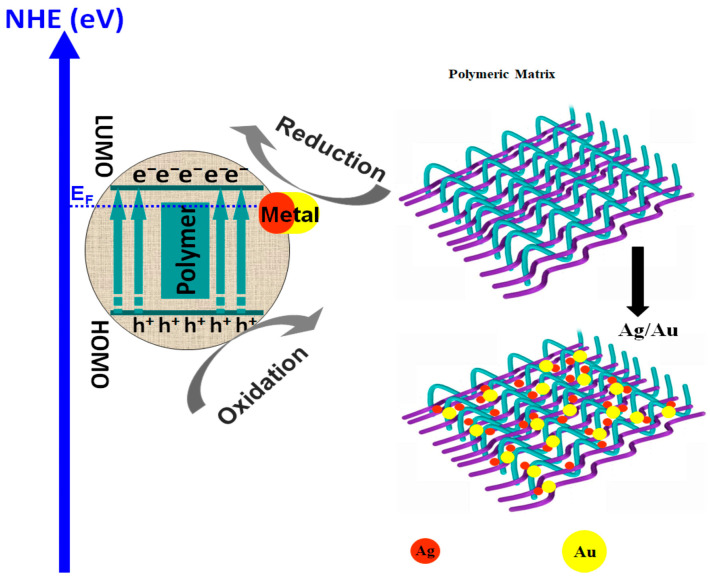
Polymer/metal composites for wastewater treatment.

Polymer/metal composites are often viewed as suitable materials for the photocatalytic removal of persistent organic pollutants, such as dyes or different pharmaceutical compounds. As presented in Figure 5, the metal nanoparticles penetrate the polymeric matrix and provide a compatible interfacial region. When conductive polymers are used, the composite materials which act as Schottky junctions represent the interface between the metal component and the semiconductor structure, providing an effective pathway to decrease the photo-generated carrier’s recombination and even to increase the catalyst spectral absorption.

The removal of methylene blue was tested using TX-SCH_2_COOH-DO/Au/Ag [79] and tris(4-carbazoyl-9-ylphenyl) amine/polyvinylpyrrolidone/Cu [80] composites. The first catalyst based on TX-SCH_2_COOH-DO/Au/Ag was tested in the presence of UV radiation provided for 90 min by a 100 W source. Using a catalyst dosage of 5 mg/5 mL, the photocatalytic efficiency reaches 72.5%. When the Au/Ag BNps composite was irradiated with UV light, due to their high surface plasmon resonance effects, a pair of electrons and holes can be developed. The electrons act to reduce adsorbed molecular oxygen on the composite surface into hydrogen peroxide radicals and superoxide radical. The photo-generated holes could follow two directions: (i) directly oxidizing the adsorbed methylene blue molecules or (ii) producing hydroxyl radicals by interacting with the water molecules or hydroxyl ions adsorbed at the surface. The second composite based on tris(4-carbazoyl-9-ylphenyl) amine/polyvinylpyrrolidone/Cu was irradiated for 90 min with a 300 W vis light source. The photocatalytic activity was 80% when the catalyst dosage was 20 mg/15 mL. If the Cu is replaced with CuO and all others parameters remain unchanged, the photocatalytic efficiency may increase at 90%. Bare CP presents good efficiency for MB removal, as a consequence of the ROS production under visible light. Photoexcited polymers will develop Coulomb-correlated electron–hole pairs, followed by interface diffusion to induce charge-separated states. The Cu presence can improve the absorption capability of the composite, while CuO can produce light harvesting and charge carrier separation.

The polyvinyl alcohol/Au/Pd composite was tested for styrene photocatalytical removal in the presence of 50 mW/cm^2^ irradiance produced by a vis source [81]. The styrene was completely removed in 60 min from the 20 mg/L initial solution concentration. It was observed that the composite catalytic effect increases with increasing light intensity until a certain point where the thermoelectron effect can damage the specific reactions. The core–shell structure of the metallic nanoparticles favors an increase in the local temperature, under visible light illumination. Consequently, the surface plasmon resonances of the localized metal nanoparticles were used to ensure a high catalytic efficiency, induced by the collective oscillations of the electrons on surfaces. High photocatalytic activity under low vis intensity radiation (9 W) was reported for phenylacetylide/Ag/Cu, which can completely remove 10 mg/L of norfloxacin in 40 min [82]. Under the same experimental conditions, the photocatalytic efficiencies decrease at 70.5% for naproxen, 64.3% for diclofenac, 47.6% for bisphenol A, and 42% for sulfisoxazole. Silver nanoparticles insertion endowed the composites with higher dispersion in aqueous solutions, which enhances their adsorption capacity as the first step in the mineralization process. The composite could not directly oxidize hydroxyl ions to radical due to its insufficient valence band potential (0.35 eV). However, the presence of •OH is due to the H_2_O_2_ conversion, which was produced by the disproportionation of •O_2_^−^ following protonation.

#### 3.2.3. Other Examples of Polymer Composite-Based Materials

Other materials, such as g-C_3_N_4_, metal sulfides, mixed metal oxides, reduced graphene oxides, etc., have been used to form polymeric-based composites with photocatalytic applications. Metal sulfides and mixed metal oxides have the advantage of larger light absorption spectra and band gap modularity based on their composition. Reduced graphene oxides and g-C_3_N_4_ may increase the active surface, providing more sites for oxidative species development. Choosing the right materials ensures a balance between the interface compatibility, light sensitivity, chemical stability, and photocatalytic performance. By coupling polypyrrole with g-C_3_N_4_, Han H. could remove 99% of methylene blue from a 10 mg/L solution [83]. The composite uses low-intensity vis light (12 W) and a dosage of 0.05 g/50 mL to generate the oxidative species. Pure g-C_3_N_4_ is a low photocatalytic material, which indicated that polypyrrole addition can enhance the active radical’s production via electron separation through the composite structure. Owing to the strong conductivity, the polypyrrole was employed as the electron’s transition channel to move onto the g-C_3_N_4_ surface, thus inhibiting the photogenerated carrier’s recombination. CdS was inserted in three different polymers (polypyrrole, polythiophene, and polyaniline) in order to study the photocatalytic removal of methylene blue from a 10 mg/L solution [84]. Due to the higher gap between the polypyrrole LUMO level and the CdS conduction band energy compared to that of polythiophene and polyaniline, the recombination rate in the polypyrrole/CdS composite was lower, resulting in better photocatalytic efficiency (77% for polypyrrole/CdS, 71% for polythiophene/CdS, and 61% for polyaniline/CdS). During irradiation with a 300 W vis source, the CdS conduction band lay below the polymer’s LUMO level (−2.0 eV for polypyrrole, −2.35 eV for polythiophene, and −2.56 eV for polyaniline). The photo-generated electrons from the polymer LUMO level were injected into the CdS conduction band and holes from the CdS valence band migrated to polymers HOMO, inducing the oxidative and reduction reactions required for oxidative species development.

**Figure 6:**
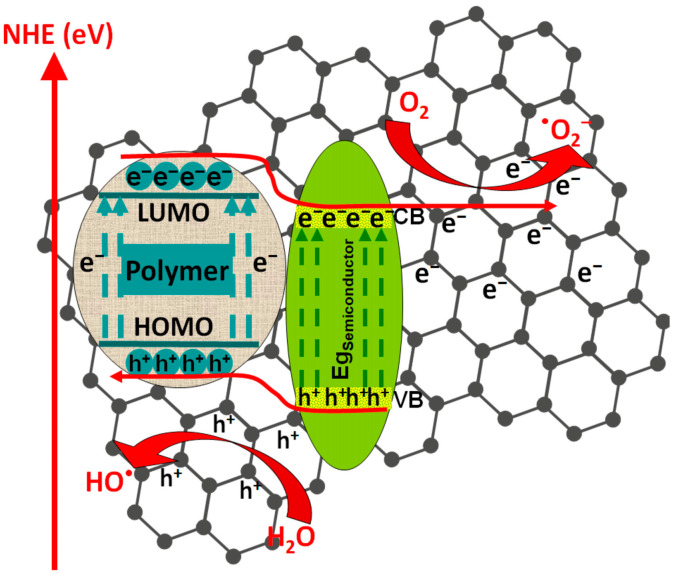
Polymer/semiconductor/GO composites with photocatalytic applications.

Polyaniline was associated with rGO/MnO_2_ [85], BiVO_4_/GO [86], and MoSe_2_ [87] in order to evaluate the composite’s photocatalytic activity in methylene blue removal. The polyaniline/rGO/MnO_2_ composite with a 10 mg/50 mL dosage could remove 90% of the 5 mg/L methylene blue solution after 120 min of irradiation with a 150 W vis source. The second composite based on polyaniline/BiVO_4_/GO (100 mg/100 mL dosage) reached a 73% removal efficiency in 180 min of irradiation with a 500 W vis source. As presented in Figure 6, both composites benefit from the graphene oxide conductive network which facilitates the charge carrier’s dispersion through the surface. The composites use the synergistic effects induced by the electron transfer from GO to polyaniline/MnO_2_ or BiVO_4_ and the limited recombination of photo-generated electron–hole pairs originating from of the ternary composition. Graphene oxide is a promising alternative for photocatalytic application due to its unique properties such as zero band gap and high surface area, as well as its ability to accept electrons in order to limit the charged carrier’s recombination. Considering the polyaniline relative energy level (π-orbital and π*-orbital) and metal oxide-GO band energy, the charges can directly migrate to the π-orbital of PANI. GO serves as the electron migration medium that facilitates the charge transfer and homogeneous metal oxide distribution in the composite. A comparative evaluation of the polyaniline/BiVO_4_/GO photocatalytic activity toward other dyes shows that the composite can remove 82% of the highly concentrated safrarine O solution (35 mg/L) and 62% of rhodamine B (4.8 mg/L) using a similar radiation scenario described for methylene blue. The third composite based on polyaniline/MoSe_2_ was tested for 120 min under 100 mW/cm^2^ vis irradiance and a 20 mg/100 mL catalyst dosage. The results indicate that 65% of methylene blue was removed due to MoSe_2_ posse’s large surface area, high chemical stability, high surface activity, and vis-NIR light-driven band gap. Additionally, 94% of methyl orange was removed by the polyaniline/MoSe_2_ composite in 150 min using the same radiation parameters. During irradiation, charge carriers’ generation took place in the polyaniline/MoSe_2_ composite due to their narrow band gap energy. The MoSe_2_ valence band and polyaniline HOMO level were positioned at 0.6 eV and 0.8 eV, favoring the hole migration from the polyaniline HOMO level to the MoSe_2_ valence band. Polyaniline was also used to form composites with LaNiSbWO_4_/GO in order to remove safrarine O (35 mg/L) and gallic acid (1.7 mg/L) under radiation produced by a 500 W vis source [88]. The sample exhibits high photocatalytic activity, reaching 84% for safrarine O and 92% for gallic acid removal in 180 min. Here, GO is considered as an electron acceptor which can enhance the charge carrier’s separation, thereby suppressing their recombination and enhancing the photocatalytic activity. In addition, the GO unpaired π electrons can interact with LaNiSbWO_4_ to increase the light absorption range.

Polyether tetraacrylate was used to form composites with Nd_0.9_TiO_3_ and LaTiO_3_ for acid black dye removal from a 15 mg/L aqueous solution [89]. The photocatalytic efficiency was similar for both composites (94% for polyether tetraacrylate /Nd_0.9_TiO_3_ and 95% for polyether tetraacrylate/LaTiO_3_) after 30 min of exposure to UV light with 250 mW/cm^2^ irradiance. Titanate perovskites are thermically stable materials with remarkable high corrosion resistance, which makes them suitable candidates for acidic environmental photocatalytic applications. The electrons located on the photocatalyst valence band (O 2p) were excited and transferred to the conduction band (Ti^4+^, empty d orbital), which react with H_2_O and dissolved oxygen molecules present in the aqueous solution to produce reactive radicals. The photodegradation of high concentrated acid orange solution (70 mg/L) was studied using the cyclodextrin/BiOBr composite with a catalyst dosage of 40 mg/40 mL [90]. The composite photocatalytic efficiency was 99.2% in 60 min of irradiation with a 500 W vis source. This result is a cumulative action of the porous cyclodextrin polymer characterized by a high specific surface, high adsorption capacities, ultrafast adsorption performances, a wide vis-light response range, and good chemical stability exhibited by BiOBr.

Polystyrene/divinylbenzene/Fe_2_O_3_ [91] and polypyrrole/Bi_2_MoO_6_ [92] composites were tested for the removal of methylene blue from an aqueous solution with similar concentration (5–8 mg/L). When the methylene blue solution was mixed with oxalic acid (38.7 mg/L), the polystyrene/divinylbenzene/Fe_2_O_3_ composite with a 10 mg/100 mL dosage reached 98% photocatalytic efficiency after 120 min of UV irradiation (20 W intensity). However, if the aqueous solution only contains oxalic acid (88.2 mg/L) as pollutant molecule, the photocatalytic efficiency may decrease at 73.6%. The composite exhibits a larger specific surface area (655 m^2^/g) due to the three-dimensional scaffold network which is beneficial for the sorption and holding of different pollutant molecules and improves the photocatalytic overall performance. The porous polymer matrix can adsorb intermediate compounds formed during photodegradation and simultaneously induce a slow release of the organic pollutant and its intermediates into the bulk solution. A similar efficiency (93.6%) was obtained by irradiating the polypyrrole/Bi_2_MoO_6_ composite with a 35 mg/50 mL dosage for 80 min with a 350 W vis light source. In the same experimental conditions, the composite reached 88.3% photocatalytic efficiency in the tetracycline solution with a 30 mg/L initial concentration. The construction of hierarchical composite photocatalysts combining the Bi_2_MoO_6_ semiconductor and the polypyrrole conductive polymer presents the advantage of uniform polymeric nanoparticle distribution on the Bi_2_MoO_6_ nanosheet surface that could effectively accelerate the photo-generated electron–hole pair’s separation. The tetracycline photocatalytic removal from a 10 mg/L solution was tested under 90 min of vis irradiation provided by a 300 W source, using 4,7-dibromobenzo thiadiazole/4-ethynylphenyl amine/Bi_2_WO_6_ as a photoactive composite [93]. The experiment was repeated with a 10 mg/L rhodamine B solution and a 20 mg/100 mL catalyst dosage. The results indicate similar photocatalytic efficiencies for tetracycline (86%) and rhodamine B (84%) due to a tight heterojunction between Bi_2_WO_6_ and the polymer, extending the light absorption range and increasing the photogenerated charge separation and transport in the heterojunction interface. The mechanism of charge photogeneration corresponds to a Z-scheme electron transfer, where the holes form a Bi_2_WO_6_ conduction band, oxidizing H_2_O to •OH radicals, while •O_2_^−^ radicals are produced by the electrons reacting with the adsorbed O_2_. The enhanced photocatalytic efficiency can be induced by the p-conjugation polymer backbone possessing unique charge separation and mobility properties.

The photocatalytic removal of erythrosine B and rose Bengal dyes was tested by irradiating the poly(trimethyl-propane triacrylate)/bis (acyl) phosphane oxide/H_3_PMo_12_O_40_ composite with 0.07 W/cm^2^ UV light [94]. After 120 min, irradiation of the composite shows 81% photocatalytic efficiency for erythrosine B removal and 86% for rose Bengal removal. When submitted to photolysis, the polymer component can generate •OH radicals in reaction with H_2_O via an oxidative hole trapping mechanism. During the photocatalytic activity, the composite can increase the number of oxidative species due to the photo-generation of simultaneous charge carriers induced by both components. A similar composite based on poly(trimethyl-propanetriacrylate)/H_3_PMo_12_O_40_/W_10_O_32_(TH)_4_ was evaluated for the removal of pharmaceutically active compounds from a 15 mg/L aqueous solution concentration [95]. The composite was irradiated with a 250 mW/cm^2^ UV–vis light source and the ibuprofen was completely removed in 90 min. However, if the irradiation time decreased at 75 min, the photocatalytic efficiency toward ciprofloxacin and oxytetracycline was 90%, and 86%, respectively. In the first step, the drugs were converted in by-products, such as hydroxylated products or other new functional group intermediates (including alcohols, olefins, and ketones). In 30 min of irradiation, the by-products were completely mineralized by the strong oxidant species generated at the composite surface. The photocatalytic degradation of the imidacloprid pesticide was investigated using a simple polyimide/WO_3_ composite as the photocatalyst [96]. The pesticide concentration was 20 mg/L and the composite was irradiated for 180 min with a 225 W vis light source. The results indicate that the photocatalytic efficiency increases from 50% to 73.2% if the catalyst dosage increases from 1 g/L to 2.5 g/L. The composite exhibits a lamellar structure with a relatively small active surface of 11.49 m^2^/g. The composite photocatalytic activity is based on the visible-light driven Z-scheme mechanism where the polymer is excited by vis light, and their band potential must be matched in order to ensure the recombination of photo-generated electrons from the polymer LUMO level and holes from the WO_3_ valence band. Then, the useful charge carriers (h^+^ from polymer HOMO level and e^−^ from WO_3_ conduction band) can participate in the oxidative species development. The insertion of phosphorus can improve the efficiency of charge separation and induce the absorption edge redshift.

## 4. Conclusions and Perspectives

This study has emerged to correlate the photocatalytic activities toward different pollutant molecules with the polymeric composite content and testing conditions. The composite photocatalytic activity depends on catalyst composition/dosage, the active surface area characteristics (active sites, pores size, and volume), the pollutant type/concentration, and the surface chemistry between the pollutant molecule and the catalyst. In processes such as adsorption, decomposition, and diffusion, the desorption kinetics of the degradation products may vary based on the chemically significantly different surfaces and composition. The paper indicates that the balance between all these factors is essential and can provide the pathway for higher photocatalytic efficiencies. Even in the situation of chemically similar materials, the presence of various crystalline forms exhibits drastically different activity. Switching from UV to vis spectra can improve the photocatalytic efficiency of the polythiophene/TiO_2_ composite toward rhodamine B from 76% to 98%. However, higher efficiency was reached with high energy costs considering the use of a 320 W vis light source for 10 h. The silver insertion in different polymeric composites induced a significant increase in the rhodamine B removal rate from 56.12% to 95%, which can be considered as a suitable method to enhance the photocatalytic activity. When pharmaceutically active compounds are used as target molecules, the dependency between the chemical compatibility of drug molecules and catalyst surfaces must be carefully evaluated. The present work has several limitations: (i) the numbers of references were reduced based on the most representative papers, (ii) several papers which may be representative were excluded due to the incomplete experimental description, and (iii) not all the parameters were considered due to the space restrictions.

For future work, the development of catalytic composites via green routes could be attempted, characterized, and compared with other commercially available nanomaterials. The green route application of composite materials is expected to add economic and scientific value to the knowledge by promoting technological development in light of the photocatalytic design. The composite materials could represent the following steps to the a large-scale industrial transition for photocatalytic degradation based on sustainable energy processes. The challenge of scaling up photocatalytic composite applications at the industrial level is one of the main issues not yet sufficiently addressed by the scientific community.

Finally, most of the studies presented in the literature target the effects of temperature, pH, pollutant concentration, or other technological parameters in relation to composite adsorption and photo-degradation capacities, whereas the problem of real wastewater pollutants is very rarely addressed. The wastewater usually contains various pollutant molecules which are ignored in most studies. The interface chemistry, absorption/desorption kinetics, and photocatalytic activity are different when complex wastewater charge pollution is considered.

## Figures and Tables

**Table 1:** Polymer/TiO_2_- or ZnO-based composites.

Composite Composition		Pollutant		Photocatalytic Properties					Ref.
Polymer (s)	Second Material	Molecule	Conc.	Radiation Type	Radiation Intensity	Exposure Time	Catalyst Dosage	Removal Efficiency	
Polyacrylamide	TiO_2_	methyl orange	1.0 mg/L	UV	3 W	300 min	np *	95%	[57]
Polyurethane	TiO_2_	methyl orange	10 mg/L	UV	38 W/m^2^	50 min	0.046 g/3.6 mL	100%	[58]
Resorcinol-formaldehyde	TiO_2_	methyl orange	13 mg/L	UV	36 W	240 min	1 mg/3 mL	55%	[59]
Polyethylene glycol	TiO_2_	methylene blue	20 mg/L	UV	300 W	240 min	3 g/100 mL	100%	[60]
Polytrifluorochloro ethylene	TiO_2_	methylene blue	10 mg/L	UV	0.5 mW/cm^2^	270 min	np	100%	[61]
PEG2000-silicone	TiO_2_	methylene blue	3.2 mg/L	UV	20 W	120 min	np	40%	[62]
Polyvinyl alcohol	TiO_2_	acid black	20 mg/L	UV	44 W/m^2^	120 min	50 mg/400 mL	55.4%	[63]
Polyethylene glycol								62.8%	
Polythiophene	TiO_2_	rhodamine B	40 mg/L	UV	10 W	180 min	300 mg/300 mL	76%	[64]
				Vis	320 W	600 min		98%	
Poly-phenylpropenes trans-anethole and N-phenylmaleimide	TiO_2_	rhodamine B	10 mg/L	UV	600 W	90 min	20 mg/20 mL	95%	[65]
		tetracycline						97%	
Chitosan Polyvinyl alcohol–chitosan	TiO_2_	metronidazole	10 mg/L	UV	32 W	120 min	0.3 g/L	100%	[66]
Polyvinyl alcohol	TiO_2_	phenol	10 mg/L	Vis	500 W	360 min	np	67.5%	[67]
Poly(vinylidene fluoride)	TiO_2_/GO	phenol	50 mg/L	UV	100 W	180 min	np	65%	[68]
Polydimethylsiloxane	TiO_2_	methylparaben	1 mg/L	Sun Radiation	np	120 min	140 mg/L	50%	[69]
		ethylparaben						52%	
		propylparaben						55%	
Polypyrrole	ZnO	methylene blue	50 mg/L	UV	100 W	20 min	50 mg/50 mL	98.12%	[70]
Polypyrrole	ZnO	rhodamine B	5 mg/L	Vis	150 W	300 min	np	65%	[71]
Poly(3-hexylthiophene-2,5-diyl)	ZnO	rhodamine B	0.01 mg/L	Vis	300 W	80 min	20 mg/100 mL	99%	[72]
Poly(propylene glycol)-dimethacrylate–methacryloyloxyethyl-N,N-dimethyl-3-(trimethoxysilyl)-propane	ZnO	methyl orange	16.35 mg/L	Vis	4.9 mW/cm^2^	250 min	1 g/50 mL	56.12%	[73]
	ZnO-Ag							95%	

* not provided.

**Table 2:** Other polymer composite-based materials.

Composite Composition		Pollutant		Photocatalytic Properties					Ref.
Polymer (s)	Second Material	Molecule	Conc.	Radiation Type	Radiation Intensity	Exposure Time	Catalyst Dosage	Removal Efficiency	
Poly(vinyl alcohol-g-acrylamide)	ZnO/SiO_2_	methylene blue	5 mg/L	UV	18 W	960 min	0.1 g/20 mL	86%	[74]
		crystal violet						77%	
		congo red						70%	
Polyaniline	Cu_2_O/ZnO	congo red	30 mg/L	UV	np *	30 min	100 mg/100 mL	100%	[75]
Polysulphone–styrene maleic anhydride copolymer	Bi_2_S_3_/TiO_2_	methylene blue	20.26 mg/L	UV–vis	350 W	180 min	np	95.32%	[76]
Polyvinyl chloride	Ag-decorated Bi_2_O_3_/Bi_2_O_2.7_	rhodamine B	12 mg/L	Vis	5 W	150 min	np	97%	[77]
Polypyrrole	TiO_2_/V_2_O_5_	tetracycline	50 mg/L	Vis	300 W	120 min	30 mg/50 mL	98%	[78]
		doxycycline						96%	
		oxytetracycline						85%	
		ofloxacin						37%	
		rhodamine B						100%	
TX-SCH2COOH-DO	Au/Ag	methylene blue	10 mg/L	UV	100 W	90 min	5 mg/5 mL	72.5%	[79]
Tris(4-carbazoyl-9-ylphenyl) amine/polyvinylpyrrolidone	Cu	methylene blue	np	Vis	300 W	90 min	20 mg/15 mL	80%	[80]
	CuO							90%	
Polyvinyl alcohol	Au/Pd	styrene	20 mg/L	Vis	50 mW/cm^2^	60 min	np	100%	[81]
Phenylacetylide	Ag/Cu	norfloxacin	10 mg/L	Vis	9 W	40 min	10 mg/50 mL	100%	[82]
		diclofenac						64.3%	
		bisphenol A						47.6%	
		naproxen						70.5%	
		sulfisoxazole						42%	
Polypyrrole	g-C_3_N_4_	methylene blue	10 mg/L	Vis	12 W	120 min	0.05 g/50 mL	99%	[83]
Polypyrrole	CdS	methylene blue	10 mg/L	Vis	75,000–90,000 Lux	300 min	25 mg/50 mL	77%	[84]
Polythiophene								71%	
Polyaniline								61%	
Polyaniline	rGO/MnO_2_	methylene blue	5 mg/L	Vis	150 W	120 min	10 mg/50 mL	90%	[85]
Polyaniline	BiVO_4_/GO	rhodamine B	4.8 mg/L	Vis	500 W	180 min	0.1 g/100 mL	62%	[86]
		methylene blue	3.2 mg/L					73%	
		safrarine O	35 mg/L					82%	
Polyaniline	MoSe_2_	methylene blue	np	Vis	100 mW/cm^2^	120 min	20 mg/100 mL	65%	[87]
		methyl orange				150 min		94%	
Polyaniline	LaNiSbWO_4_/GO	safrarine O	35 mg/L	Vis	500 W	180 min	0.1 g/100 mL	84%	[88]
		gallic acid	1.7 mg/L					92%	
Polyether Tetraacrylate	Nd_0.9_TiO_3_	Acid Black	15 mg/L	UV	250 mW/cm^2^	30 min	np	94%	[89]
	LaTiO_3_							95%	
Cyclodextrin	BiOBr	Acid Orange 7	70 mg/L	Vis	500 W	60 min	40 mg/40 mL	99.2%	[90]
Polystyrene/divinylbenzene	Fe_2_O_3_	methylene blue + oxalic acid	8 mg/L + 38.7 mg/L	UV	20 W	120 min	10 mg/100 mL	98%	[91]
		oxalic acid	88.2 mg/L					73.6%	
Polypyrrole	Bi_2_MoO_6_	methylene blue	5 mg/L	Vis	350 W	80 min	35 mg/50 mL	93.6%	[92]
		tetracycline	30 mg/L					88.3%	
4,7-dibromobenzo thiadiazole/4-ethynylphenyl amine	Bi_2_WO_6_	tetracycline	10 mg/L	Vis	300 W	90 min	20 mg/100 mL	86%	[93]
		rhodamine B						84%	
Poly(trimethyl-propane triacrylate)/bis(acyl)phosphane oxides	H_3_PMo_12_O_40_	erythrosine B	10 mg/L	UV	0.07 W/cm^2^	120 min	np	81%	[94]
		rose bengal						86%	
Poly(trimethyl-propane triacrylate)	H_3_PMo_12_O_40_ W_10_O_32_ (TH)_4_	ibuprofen	15 mg/L	UV–vis	250 mW/cm^2^	90 min	np	100%	[95]
		ciprofloxacin				75 min		90%	
		oxytetracycline				75 min		86%	
Polyimide	WO_3_	imidacloprid	20 mg/L	Vis	225 W	180 min	1 g/L	50%	[96]
							2.5 g/L	73.2%	

* not provided.

## Data Availability

Data presented in this study are available upon request from the corresponding author.
